# Extracellular Matrix-Based Gene Expression Signature Defines Two Prognostic Subtypes of Hepatocellular Carcinoma With Different Immune Microenvironment Characteristics

**DOI:** 10.3389/fmolb.2022.839806

**Published:** 2022-03-25

**Authors:** Hui Tang, Tingting You, Zhao Sun, Chunmei Bai, Yingyi Wang

**Affiliations:** Department of Medical Oncology, Peking Union Medical College Hospital, Chinese Academy of Medical Sciences and Peking Union Medical College, Beijing, China

**Keywords:** liver cancer, extracellular matrix, molecular subtype, tumor-infiltrating immune cell, risk score, prognostic evaluation, drug sensitivity

## Abstract

**Background:** Accumulating evidence has suggested that the extracellular matrix (ECM) plays a vital role in the development and progression of cancer, and could be recognized as a biomarker of the response to immunotherapy. However, the effect of the ECM signature in hepatocellular carcinoma (HCC) is not well understood.

**Methods:** HCC patients derived from the TCGA-LIHC dataset were clustered according to the ECM signature. The differences in prognosis, functional enrichment, immune infiltration, and mutation characteristics between distinct molecular clusters were examined, and its predictive value on the sensitivities to chemotherapy and immunotherapy was further analyzed. Then, a prognostic model was built based on the ECM-related gene expression pattern.

**Results:** HCC patients were assigned into two molecular subtypes. Approximately 80% of HCC patients were classified into cluster A with poor prognosis, more frequent TP53 mutation, and lower response rate to immunotherapy. In contrast, patients in cluster B had better survival outcomes and higher infiltration levels of dendritic cells, macrophages, and regulatory T cells. The prognostic risk score model based on the expression profiles of six ECM-related genes (SPP1, ADAMTS5, MMP1, BSG, LAMA2, and CDH1) demonstrated a significant association with higher histologic grade and advanced TNM stage. Moreover, the prognostic risk score showed good performance in both the training dataset and validation dataset, as well as improved prognostic capacity compared with TNM stage.

**Conclusions:** We characterized two HCC subtypes with distinct clinical outcomes, immune infiltration, and mutation characteristics. A novel prognostic model based on the ECM signature was further developed, which may contribute to individualized prognostic prediction and aid in clinical decision-making.

## Introduction

Hepatocellular carcinoma (HCC) accounts for approximately 80% of liver cancers and is the fourth leading cause of cancer-related death worldwide (Bray et al., 2018). Unfortunately, although surgery may effect a radical cure, as many as 70% of HCC patients would have tumor recurrence after surgery at 5 years (Villanueva, 2019). With an estimated survival time of approximately 1 year, sorafenib and lenvatinib remained the only effective systemic therapies for frontline therapy until immune checkpoint inhibitor (ICI)-based combination therapy showed desirable efficacy, but clinically available biomarkers to predict response to systemic therapies are still needed (Villanueva, 2019; Pinter et al., 2021). Therefore, it is urgent to further explore the underlying mechanisms of cancer development and detect novel prognostic and therapeutic targets of HCC.

Most biomarkers used for cancer classification and stratification are still “cancer-cell oriented” (such as TNM stage and tumor markers), leaving out key factors associated with cancer development, such as the tumor microenvironment (TME) and antitumor immunity (Lecchi et al., 2021). The TME is composed of three interrelated components, including stromal cells such as immune cells and fibroblasts, cytokines, and the extracellular matrix (ECM) (Théret et al., 2021). Previous studies have proven that HCC classification based on molecular features of immune infiltration could aid in prognosis evaluation and predicting response to ICIs (Sia et al., 2017; Kurebayashi et al., 2018). Accumulating evidence has suggested that the ECM is associated with tumor aggression, metastasis, treatment sensitivity, and prognosis (Deligne and Midwood, 2021). The ECM was also reported to not only provide a physical barrier, preventing interaction between immune effectors or drugs and tumor cells, but can also modulate immune cell proliferation, differentiation, motility, and activation (Zhang et al., 2021). However, the role of the ECM signature in HCC classification has not been estimated, although a dynamic ECM was associated with HCC carcinogenesis, progression, and prognosis (Jeng et al., 2015).

In the present research, we first classified patients from The Cancer Genome Atlas liver hepatocellular carcinoma (TCGA-LIHC) cohort into distinct molecular subtypes according to ECM-related gene expression. The relationships between molecular subtypes and clinicopathological features, prognosis, and drug sensitivity were further examined. Then, an ECM-related prognostic signature was developed and validated.

## Materials and Methods

### Data Sources

Gene expression, somatic mutation, and corresponding clinicopathological data of 369 HCC samples were obtained from TCGA database (https://portal.gdc.cancer.gov/) in December 2021. One International Cancer Genome Consortium (ICGC) (https://dcc.icgc.org/) and two Gene Expression Omnibus (GEO) (https://www.ncbi.nlm.nih.gov/geo/) HCC cohorts (there were 232, 80, and 81 HCC patients with complete follow-up information in ICGC-LIRI, GSE10141, and GSE76427 cohorts, respectively) were obtained for external validation. The gene expression values were transformed into transcripts per kilobase million (TPM) (Wagner et al., 2012). The mean value was reserved if one gene matched multiple probes. The ComBat method was used to remove the batch effects.

### Consensus Clustering Analysis

Three hundred and one ECM-related genes were retrieved from Reactome (https://reactome.org/) (Jassal et al., 2020). Consensus unsupervised clustering analysis was conducted by the R package “ConsensusClusterPlus” (Wilkerson and Hayes, 2010) (1,000 iterations, resample rate of 80%) to classify patients from the TCGA-LIHC cohort into distinct molecular subtypes according to ECM-related gene expression. This clustering was conducted, and the optimal cluster number was confirmed based on the following criteria: First, the cumulative distribution function (CDF) curve increased gradually and smoothly. Second, after clustering, the intracluster correlation increased, while the intercluster correlation decreased. Last, no clusters had a too small sample size.

### Exploring the Differences Between Distinct Molecular Clusters of HCC Patients

The differences in overall survival (OS) among different subtypes were assessed using Kaplan–Meier curves generated by the R packages “survival” and “survminer”. The top 20 highest mutational frequencies in the TCGA-LIHC cohort were recognized and visualized via the R package “maftools”. Tumor mutation burden (TMB) was defined as the total gene mutation number per million base pair, which was calculated using a Perl script and corrected by dividing by the total length of exons. The immune cell infiltration abundance data in the TCGA database were retrieved from xCell (https://xcell.ucsf.edu/) (Aran et al., 2017), and the CIBERSORT algorithm was used to evaluate the immune and stromal scores of each patient (Newman et al., 2015). xCell is a transcriptome-based method learned from thousands of pure cell types from various sources that performs cell type enrichment analysis from gene expression data for 64 immune cell types (Aran et al., 2017). Furthermore, gene set enrichment analysis (GSEA) was conducted to investigate the functional enrichment of differentially expressed genes in distinct molecular clusters using the R packages “limma”, “org.Hs.eg.db”, “clusterProfiler”, and “enrichplot”. Gene ontology (GO) and Kyoto Encyclopedia of Genes and Genomes (KEGG) gene sets (c5.all.v7.1 and c2.cp.kegg.v7.1) were downloaded from the MSigDB database (https://www.gsea-msigdb.org/gsea/index.jsp).

### Drug Sensitivity Analysis

The half-maximal inhibitory concentration (IC50) of several chemotherapy or targeted drugs in each HCC sample from TCGA database was estimated via Genomics of Drug Sensitivity in Cancer (GDSC; https://www.cancerrxgene.org/) (Yang et al., 2013) using the R package “pRRophetic” (Geeleher et al., 2014), which is extensively utilized in studies evaluating drug sensitivity in cancers (Xiao et al., 2021; Liu et al., 2021a). Each sample’s IC50 value was evaluated by ridge regression, and 10-fold cross-validation was used to ensure prediction accuracy based on the GDSC training model. The Tumor Immune Dysfunction and Exclusion (TIDE) (https://tide.dfci.harvard.edu/) algorithm was implemented to predict the ICI therapy response of each patient (Jiang et al., 2018). TIDE is a prevalent algorithm used to assess immune evasion mechanism and predict the immunotherapeutic response (Jiang et al., 2018; Liu et al., 2021b). TIDE scores and estimated immunotherapeutic responses were obtained after uploading the input data as described in the instructions, and higher TIDE scores mean a lower likelihood of response to immunotherapy.

### Construction of an ECM-Related Prognostic Signature

Least absolute shrinkage and selection operator (LASSO) regression analysis with the R package “glmnet” was used to identify the candidate genes of the ECM-related prognostic signature. Based on the common genes and corresponding expression profiles in the four independent cohorts mentioned above, six ECM-related genes were selected to build the risk signature according to the optimal lambda value and the corresponding coefficients using the TCGA-LIHC dataset. The risk score of the prognostic signature for each patient was calculated as follows: risk score = (exp Gene1 × coef Gene1) + (exp Gene2 × coef Gene2) + … + (exp GeneN × coef GeneN).

### Validation of the ECM-Related Prognostic Signature

Patients were divided into two risk groups (low/high) according to their risk score and based on the optimal cutoff value of OS calculated by the R package “survminer”. Propensity-score matching (PSM) was used in the TCGA-LIHC dataset to minimize the impact of confounding factors. The propensity scores were calculated by the R package “MatchIt” using multivariable logistic regression based on age, TNM stage, histologic grade, Child–Pugh grade, vascular invasion, alpha fetoprotein, and residual tumor after surgery. Then, the risk scores of each patient in three validation cohorts were calculated and divided using the same formula and optimal cutoff value. The OS of the two risk score groups was compared using Kaplan–Meier curves. Univariate and multivariate Cox proportional hazard models were performed by the R package “finalfit” to calculate the hazard ratios (HR) with 95% confidence intervals (CI) of variables associated with OS in HCC patients. Based on the results of multivariate Cox analysis, a nomogram was constructed by the R package “rms” and assessed by the receiver operating characteristic (ROC) curve, concordance index (C-index), and calibration curves. Ultimately, decision curve analysis (DCA) was performed by the R package “rmda” to compare the prognostic capacity of the nomogram model and TNM stage.

### Statistical Analysis

The Mann–Whitney *U* test and Pearson’s chi-square test or Fisher’s exact test were applied to the comparison of the difference between the continuous and categorical variables between two groups, respectively. Strawberry Perl (version 5.30.0, https://strawberryperl.com/) was used to extract gene expression and mutation data from downloaded datasets and transfer them into a data matrix for subsequent analyses. All statistical analyses and visualization were performed using R software (version 3.6.1, https://www.r-project.org/). A two-tailed value of P or FDR (false discovery rate) q < 0.05 was considered to be statistically significant.

## Result

### Consensus Clustering of ECM-Related Genes Identified two Clusters of HCC

**FIGURE 1:**
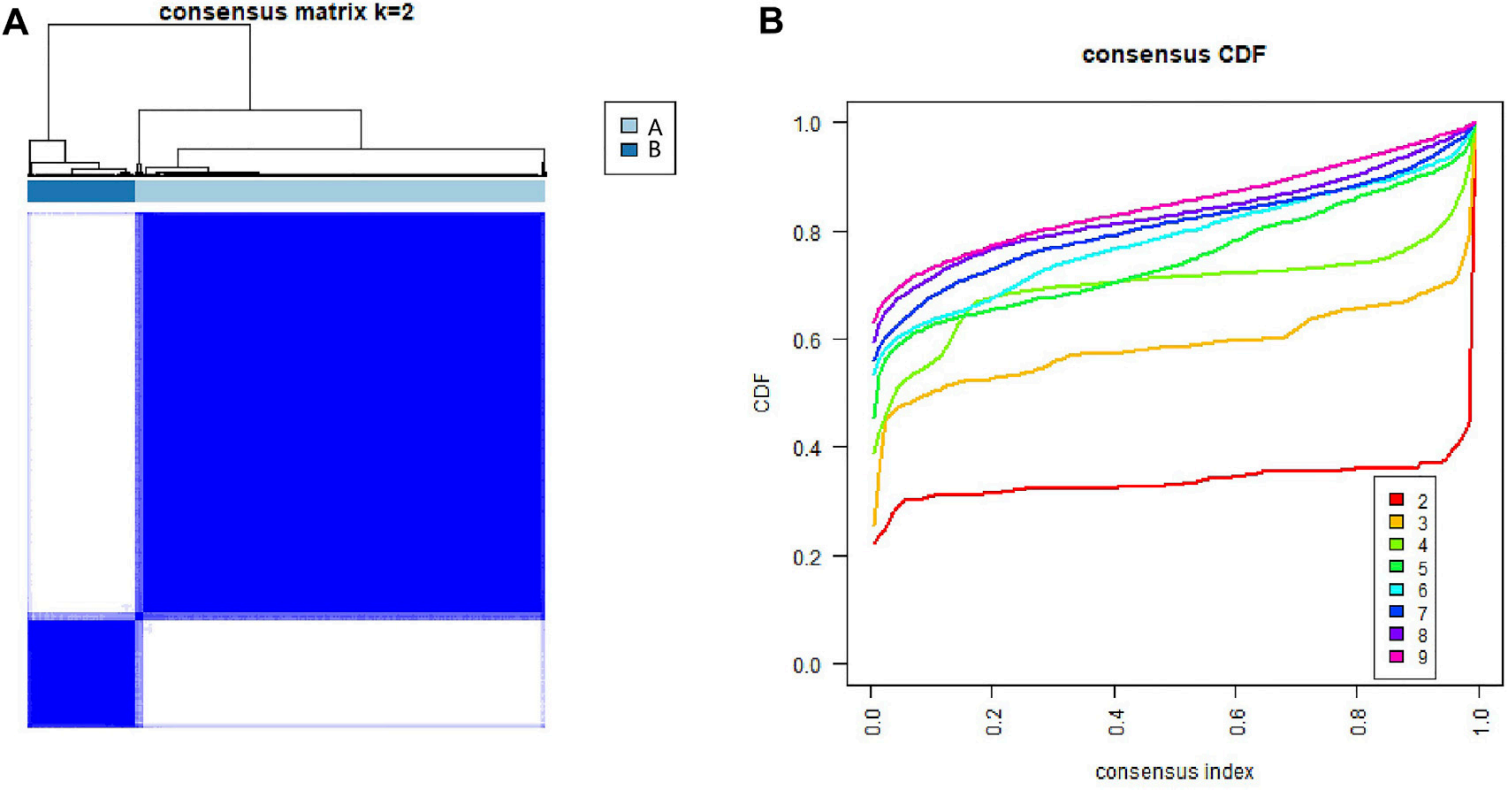
Identification of consensus clusters of extracellular matrix-related genes in hepatocellular carcinoma from the TCGA database. **(A)** Consensus clustering matrix for k = 2. **(B)** Consensus clustering CDF for k = 2–9. CDF, cumulative distribution function.

To examine the roles of ECM-related genes in HCC, 371 patients with transcriptome data who were retrieved from the TCGA-LIHC dataset were categorized using a consensus clustering algorithm based on the expression profiles of 301 ECM-related genes. Unsupervised consensus clustering analysis indicated that k = 2 was the optimal selection; thus, patients in the entire cohort were sorted into subtypes A (*n* = 294, 79.2%) and B (*n* = 77, 20.8%) (Figures 1A,B).

### Relationship Between Molecular Subtypes and Clinicopathological Features and Prognosis

**FIGURE 2:**
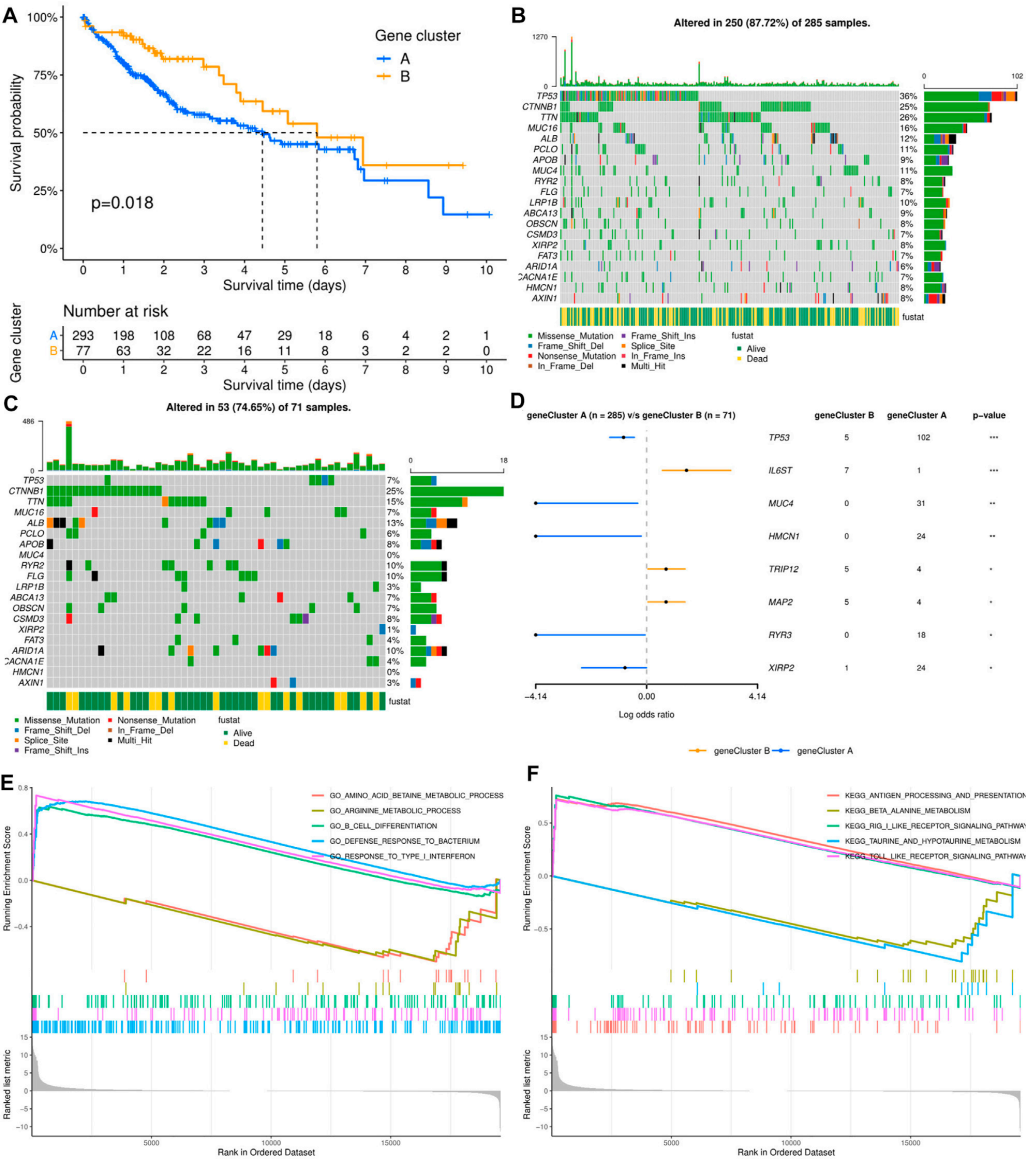
Relationship between molecular subtypes and clinicopathological features and prognosis. **(A)** Kaplan–Meier survival curves of the two molecular subtypes. **(B, C)** Distribution and phenotype of the top 20 gene mutations in clusters **(A, B)**. **(D)** Analysis of the frequency difference of gene mutations in the two molecular subtypes. **(E, F)** GO and KEGG analysis of enriched biological pathways in cluster **(A)** versus cluster **(B)**.

As shown in Figure 2A, the results of survival analysis showed that the patients’ prognosis was significantly different between the two subtypes. This suggested that there may be certain distinct differences apart from clinical outcomes between the two subtypes. However, there was no significant difference in clinical characteristics between the two molecular subtypes, including age, sex, TNM stage, histologic grade, Ishak score, Child–Pugh grade, and vascular invasion (Supplementary Table S1). To further explore the differences between the two molecular subgroups, three hundred and sixty-one HCC patients with somatic mutation data were included for mutational feature analysis. Mutation profile features indicated that missense mutation was the most common type in both molecular subtypes (Figures 2B,C). The top 20 mutated genes were visualized by a horizontal histogram, and the results showed that TP53 (35.8 *vs.* 7.0%), MUC4 (10.9 *vs.* 0%), XIRP2 (8.4 *vs.* 1.4%), HMCN1 (8.4*vs.*vs 0%), and RYR3 (6.3 *vs.* 0%) mutations were more frequent in cluster A, while IL6ST (0.4 *vs.* 9.9%), TRIP12 (1.4*vs.* 7.0%), and MAP2 (1.4 *vs.* 7.0%) mutations were more frequent in cluster B (Figure 2D). Furthermore, mutant CTNNB1, TTN, and ALB were the most common mutations in both subtypes.

To elucidate the potential influence of the ECM-related subtypes on the expression profiles of HCC, GSEA was applied to compare clusters A and B. Functional enrichment analysis showed that immune-related pathway terms (GO_B_CELL_DIFFERENTIATION, GO_DEFENSE_RESPONSE_TO_ BACTERI-UM, GO_RESPONSE_TO_TYPE_I_INTERFERON, KEGG_ANTIGEN_PROCES-SING_AND_PRESENTATION, KEGG_RIG_I_LIKE_RECEPTOR_SIGNALING_ PATHWAY, and KEGG_TOLL_LIKE_RECEPTOR_SIGNALING_PATHWAY) were significantly enriched in cluster A, while some pathway terms relevant to amino acid metabolism were significantly enriched in cluster B (GO_AMINO_ACID_BETAINE_ METABOLIC_PROCESS, GO_ARGININE_METABOLIC_PROCESS, KEGG_BE-TA_ALANINE_METABOLISM, and KEGG_TAURINE_AND_HYPOTAURINE_ METABOLISM) (Figures 2E,F).

**FIGURE 3:**
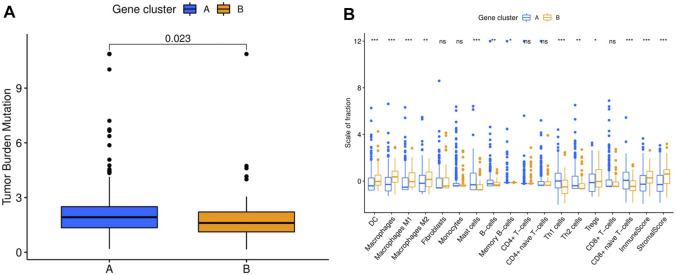
Comparison of TMB and immune cell infiltration abundance between two hepatocellular carcinoma molecular subtypes. **(A)** TMB in different molecular subtypes. **(B)** Immune cell infiltration abundance in different molecular subtypes. TMB, tumor mutation burden. *p < 0.05, **p < 0.01, ***p < 0.001.

ICI-based combination therapy is one of the most important breakthroughs in the treatment of HCC in recent years, while TMB and tumor-infiltrating immune cells may be potential biomarkers to predict the response to ICIs (Pinter et al., 2021). Therefore, we examined the differences in TMB and immune microenvironment between the two molecular subtypes. For TMB, the results showed that patients in cluster A had significantly higher TMB, but almost all HCC patients derived from TCGA database had low TMB (<10 mutations per megabase) (Figure 3A). In contrast, patients in cluster B had higher immune and stromal scores. Furthermore, the infiltration levels of dendritic cells (DCs), macrophages, M1 and M2 macrophages, and regulatory T cells (Tregs) were significantly higher in cluster B, while mast cells, B cells, memory B cells, Th1 and Th2 cells, and CD8^+^ naive T cells had significantly lower infiltration in cluster B than in cluster A. In addition, there was no significant difference in the infiltration levels of fibroblasts, monocytes, CD4^+^ T cells, and CD8^+^ T cells between the two molecular subtypes (Figure 3B).

### Drug Sensitivity Estimation

**FIGURE 4:**
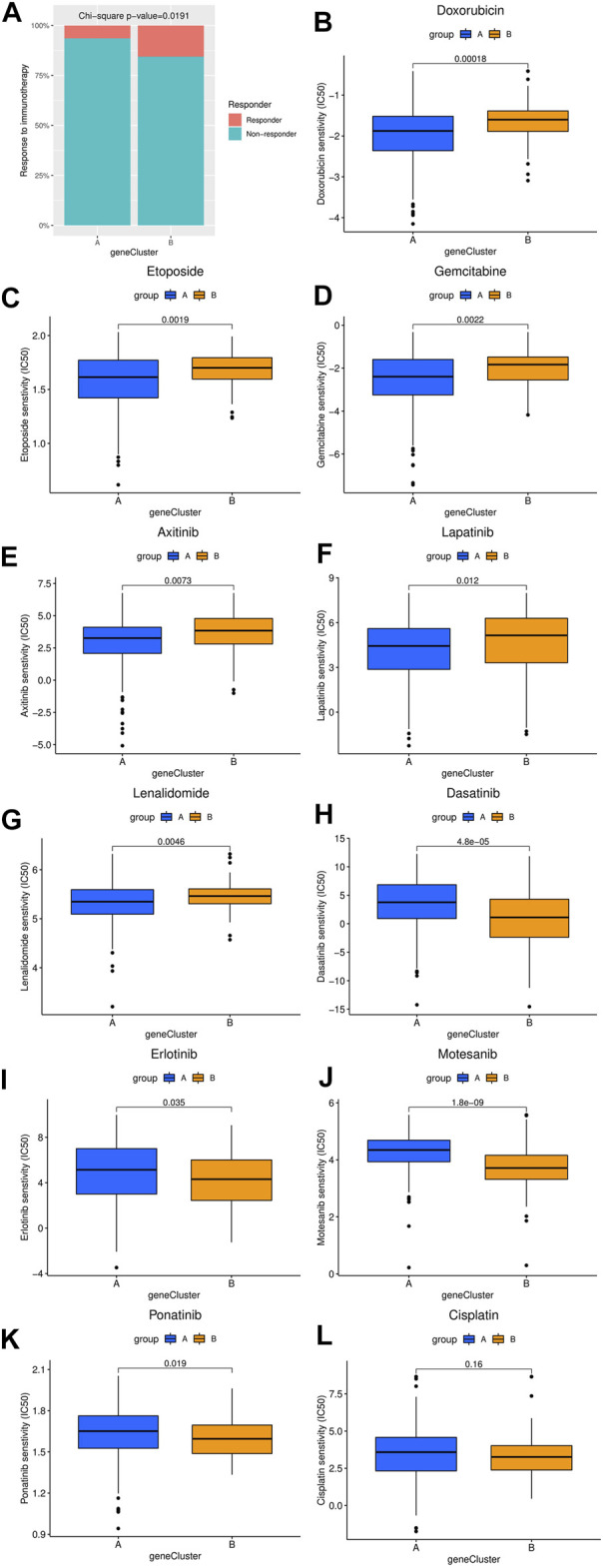
Comparison of the difference in estimated drug sensitivity between two hepatocellular carcinoma molecular subtypes. **(A)** The response to immunotherapy estimated by the TIDE database in different molecular subtypes. **(B–L)** Estimated IC50 values of chemotherapy or targeted therapy drugs in the two molecular subtypes. The higher the sensitivity to the drug, the lower the IC50 value. IC50, half maximal inhibitory concentration.

The differences in the sensitivity of immunotherapy, several chemotherapy and targeted therapy drugs between the two HCC clusters were analyzed using the TIDE and GDSC databases. As shown in Figure 4A, the response rate to ICIs predicted by the TIDE database was significantly higher in cluster B patients. Furthermore, the estimated IC50 values of doxorubicin, etoposide, gemcitabine, axitinib, lapatinib, and lenalidomide were significantly lower in cluster A samples than in cluster B samples, while the estimated IC50 values of dasatinib, erlotinib, motesanib, and ponatinib were significantly lower in cluster B samples (Figures 4B–K). However, the estimated IC50 value of cisplatin was not significantly different between clusters A and B (Figure 4L).

### Development and Validation of the ECM-Related Prognostic Signature

**FIGURE 5:**
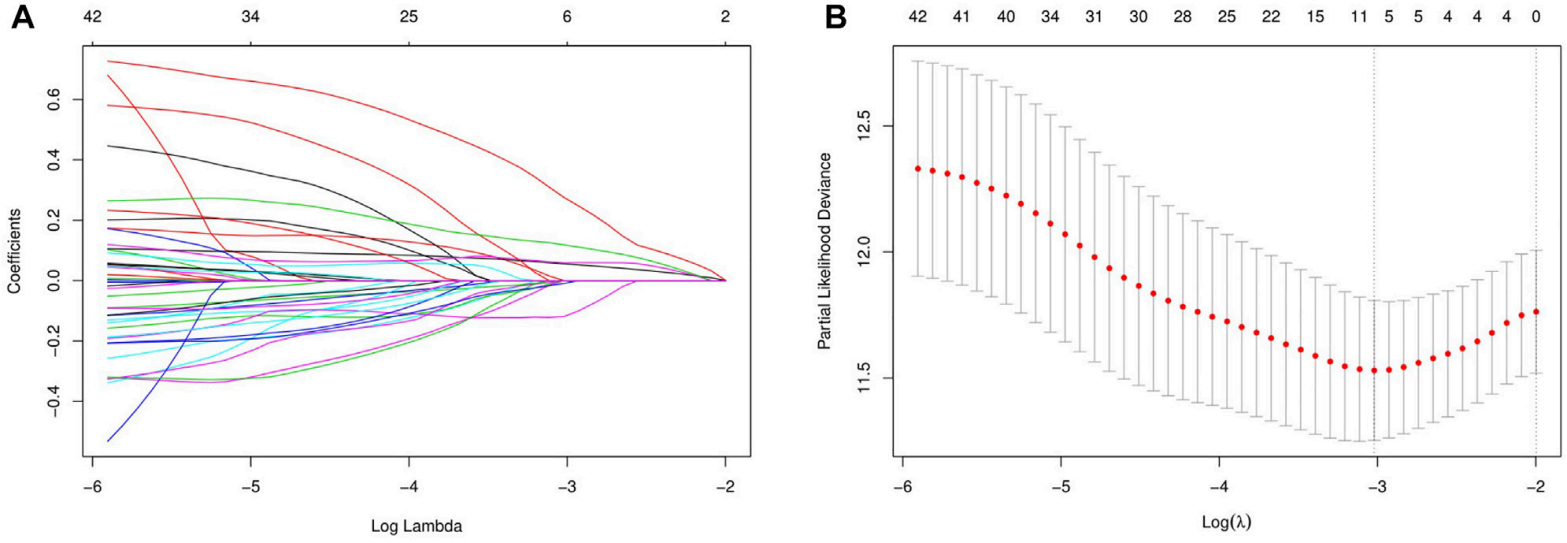
Identification of the extracellular matrix-related prognostic signature. **(A)** LASSO coefficient profiles of the prognostic genes. **(B)** Parameter selection in the LASSO model. LASSO, least absolute shrinkage and selection operator.

**FIGURE 6:**
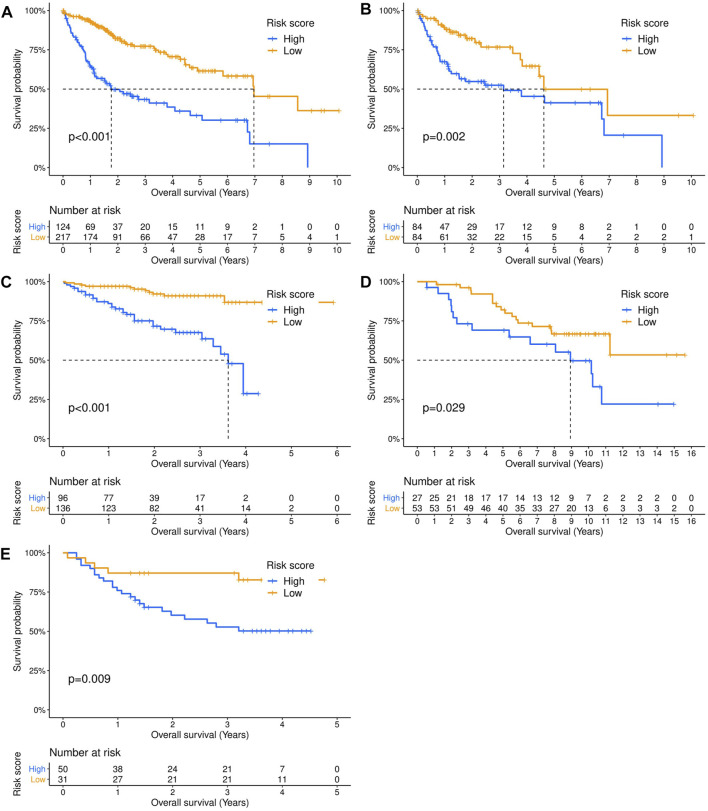
Validation of the prognostic value of the extracellular matrix-related prognostic signature in different datasets. The Kaplan–Meier survival curves showed the overall survival outcomes of patients from a dataset who were stratified into two groups according to the optimal cutoff values for the risk scores. **(A)** TCGA-LIHC dataset. **(B)** TCGA-LIHC dataset (adjusted after propensity score matching analysis). **(C)** ICGC-LIRI dataset. **(D)** GSE10141 dataset. **(E)** GSE76427 dataset.

Three hundred forty-one HCC patients with complete TNM stage and follow-up information were used for the development of an ECM-related prognostic signature. Following LASSO regression analysis, six genes remained according to the minimum partial likelihood deviance (Figures 5A,B). According to the results of LASSO regression, the ECM-related prognostic risk score was constructed as follows: Risk score = (0.055493666* expression of SPP1) + (0.275597331* expression of ADAMTS5) + (0.058747561* expression of MMP1) + (0.118350341* expression of BSG) + (-0.119029579* expression of LAMA2) + (-0.009262772* expression of CDH1). The risk score of each patient was calculated using this formula. According to the optimal cutoff value of the risk score, patients were divided into high- and low-risk groups. As shown in Table 1, the risk score was not significantly associated with sex, Ishak score, alpha fetoprotein (AFP), or residual tumor status. However, the high-risk group was significantly correlated with higher histologic grade, advanced age and TNM stage, indicating that a higher risk score may be correlated with HCC progression. Kaplan–Meier survival analyses showed that high-risk patients had remarkably reduced OS compared with low-risk patients in the TCGA-LIHC dataset, even if confounding factors (age, TNM stage, histologic grade, Child–Pugh grade, vascular invasion, AFP, and residual tumor status after surgery) were adjusted after PSM analysis (Figures 6A,B). Furthermore, the prognostic value of the risk score was robust when validated in three external validation datasets (Figures 6C–E).

**TABLE 1:** Association between risk score and the clinicopathological variables in HCC patients (*n* = 341).

	Risk group			*p*
	Total	High	Low	
	(N = 341)	(N = 125)	(N = 216)	
Age				
<60	159 (46.6%)	49 (39.2%)	110 (50.9%)	**0.048**
≥60	182 (53.4%)	76 (60.8%)	106 (49.1%)	
Gender				
Female	109 (32.0%)	45 (36.0%)	64 (29.6%)	0.273
Male	232 (68.0%)	80 (64.0%)	152 (70.4%)	
Family history of cancer				
No	196 (57.5%)	69 (55.2%)	127 (58.8%)	0.214
Yes	100 (29.3%)	43 (34.4%)	57 (26.4%)	
Unknown	45 (13.2%)	13 (10.4%)	32 (14.8%)	
TNM stage				
I	170 (49.9%)	44 (35.2%)	126 (58.3%)	**<0.001**
II	84 (24.6%)	35 (28.0%)	49 (22.7%)	
III	83 (24.3%)	45 (36.0%)	38 (17.6%)	
IV	4 (1.2%)	1 (0.8%)	3 (1.4%)	
Histologic grade				
G1–G2	212 (62.2%)	69 (55.2%)	143 (66.2%)	**0.032**
G3–G4	127 (37.2%)	54 (43.2%)	73 (33.8%)	
Unknown	2 (0.6%)	2 (1.6%)	0 (0%)	
Ishak score				
0–4	124 (36.4%)	39 (31.2%)	85 (39.4%)	0.253
5–6	74 (21.7%)	27 (21.6%)	47 (21.8%)	
Unknown	143 (41.9%)	59 (47.2%)	84 (38.9%)	
Child–Pugh grade				
A	207 (60.7%)	60 (48.0%)	147 (68.1%)	**0.001**
B-C	21 (6.2%)	9 (7.2%)	12 (5.6%)	
Unknown	113 (33.1%)	56 (44.8%)	57 (26.4%)	
Vascular invasion				
Macro	16 (4.7%)	9 (7.2%)	7 (3.2%)	**0.001**
Micro	84 (24.6%)	33 (26.4%)	51 (23.6%)	
None	193 (56.6%)	56 (44.8%)	137 (63.4%)	
Unknown	48 (14.1%)	27 (21.6%)	21 (9.7%)	
Alpha fetoprotein				
Negative	87 (25.5%)	24 (19.2%)	63 (29.2%)	0.057
Positive	254 (74.5%)	101 (80.8%)	153 (70.8%)	
Residual tumor				
R0	301 (88.3%)	105 (84.0%)	196 (90.7%)	0.068
R1-R2	14 (4.1%)	5 (4.0%)	9 (4.2%)	
Unknown	26 (7.6%)	15 (12.0%)	11 (5.1%)	

The bold values indicate they are statistically significant (*p* < 0.05).

### Construction and Evaluation of the ECM-Related Prognostic Nomogram

**FIGURE 7:**
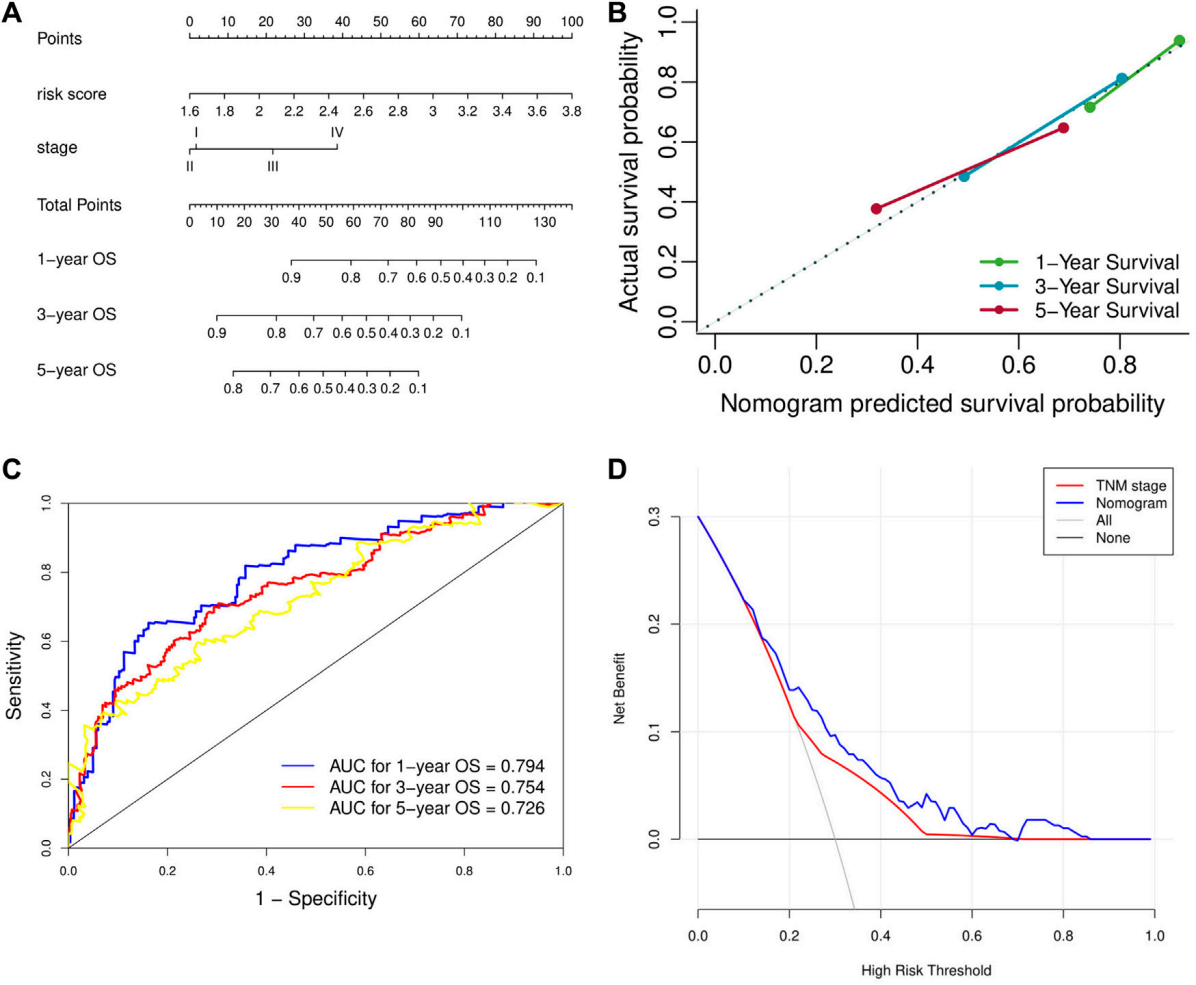
Validation of the prognostic value of the extracellular matrix-related prognostic signature based on the nomogram. **(A)** The nomogram predicting overall survival. **(B)** The calibration curve of the nomogram. **(C)** The ROC curve of the nomogram. **(D)** The DCA curve of the nomogram. ROC, receiver operating characteristic. DCA, decision curve analysis.

Univariate analysis demonstrated that risk score, vascular invasion status, and TNM stage were significantly associated with the OS of HCC patients. Further multivariate analysis confirmed that a higher risk score (HR: 4.45, 95% CI: 2.75–7.23, *p* < 0.001) and advanced TNM stage were independent indicators of unfavorable OS (Table 2). According to the results of multivariate Cox analyses, a nomogram predicting the OS of HCC patients was constructed based on TNM stage and risk score (Figure 7A). The C-index of the nomogram for OS prediction was 0.722 (95% CI: 0.697–0.748). As shown in the calibration plot (Figure 7B), the nomogram demonstrated excellent agreement between the predicted and actual survival outcomes (1-, 3-, and 5-years OS) after surgery. The areas under the curve (AUC) for 1-, 3-, and 5-years OS were 0.794, 0.754, and 0.726, respectively (Figure 7C). In addition, the DCA curve demonstrated that the signature–stage nomogram showed better prognostic capacity than TNM stage (Figure 7D).

**TABLE 2:** Univariate and multivariate analyses of overall survival.

Variables	Univariate analysis		Multivariate analysis	
	HR (95% CI)	*p*	HR (95% CI)	*p*
Age (≥ 60 *vs.* < 60)	1.25(0.87,1.81)	0.234	−	−
Gender (Female *vs.* Male)	1.28(0.88,1.86)	0.197	−	−
Family history of cancer (Yes *vs.* No)	1.13(0.77,1.68)	0.532	−	−
TNM stage (II *vs.* I)	1.42(0.87,2.32)	0.158	1.02(0.57,1.82)	0.944
TNM stage (III *vs.* I)	2.68(1.75,4.08)	**<0.001**	1.76(1.07,2.88)	**0.025**
TNM stage (IV *vs.* I)	5.5(1.7,17.82)	**0.005**	3.84(1.17,12.57)	**0.026**
Histologic grade (G3–G4 *vs.* G1–G2)	1.14(0.79,1.67)	0.481	−	−
Ishak score (5–6 *vs.* 0–4)	0.87(0.5,1.5)	0.613	−	−
Child–Pugh grade (B–C *vs.* A)	1.66(0.82,3.36)	0.159	−	−
Vascular invasion (Micro *vs.* None)	0.51(0.23,1.14)	0.101	0.77(0.33,1.82)	0.556
Vascular invasion (Macro *vs.* None)	0.44(0.21,0.93)	**0.032**	0.87(0.39,1.93)	0.735
Alpha fetoprotein (Positive *vs.* Negative)	1.2(0.78,1.83)	0.412	−	−
Residual tumor (R1–R2 *vs.* R0)	1.16(0.47,2.86)	0.74	−	−
Risk score (High *vs.* Low)	5.29(3.35,8.35)	**<0.001**	4.45(2.75,7.23)	**<0.001**

The bold values indicate they are statistically significant (*p* < 0.05).

## Discussion

With advancements in high-throughput sequencing technology, accumulated prognostic biomarkers and therapeutic targets have been identified and have promoted our understanding of cancer. Previous studies have proven that the ECM-related signature is associated with prognosis and the immune microenvironment in breast cancer and esophageal cancer (Lecchi et al., 2021; Zhang et al., 2021). However, reliable biomarkers for the immunotherapy response and prognosis in HCC based on the ECM are still very rare. Considering the influence of ECM alteration in the TME shares a common molecular mechanism in carcinogenesis and progression, it can be regarded as a pancancer effect (Yu et al., 2021). In addition, liver cirrhosis is closely related to liver cancer, which is also a well-known pathological condition linked to ECM stiffening (Piersma et al., 2020). It is understandable that the ECM plays an important role in HCC.

In the current study, using data from the TCGA-LIHC dataset, we first found that HCC patients could be categorized into two subtypes by the expression profile of ECM-related genes. Moreover, there was a significant difference in survival outcome between the two molecular subtypes, which confirmed the role of the ECM in HCC prognosis. However, it is difficult to speculate which subtype a patient belongs to based only on clinical features. Furthermore, mutation characteristics analysis revealed that cluster A, with poor prognosis and higher TMB, had higher mutation frequencies of TP53, MUC4, XIRP2, HMCN1, and RYR3, while cluster B had higher mutation frequencies of IL6ST, TRIP12, and MAP2. This is consistent with previous reports that mutant MUC4 was correlated with higher TMB and potentially associated with prognosis in pancancer (Yang Y. et al., 2020), while XIRP2 mutation was potentially associated with metastasis in breast cancer (Paul et al., 2020). Previous studies have reported that genetic mutations in HCC have something to do with risk factors and centered on CTNNB1 (alcohol) and TP53 (HBV) (Schulze et al., 2015), which may be one of the underlying mechanisms of grouping. In addition, mutant TP53 has been consistently associated with poor prognosis in a wide variety of cancers, including HCC (Olivier et al., 2010; Villanueva, 2019). IL6ST is recognized as an oncogene involved in tumorigenesis and associated with inflammatory hepatocellular adenomas (Sun et al., 2014). Functional enrichment analysis demonstrated that immune-relevant pathways were significantly enriched in cluster A, while some pathways related to amino acid metabolism were significantly enriched in cluster B. This is consistent with a previous study that the HCC subgroup with enriched amino acid metabolism-relevant pathways showed a good prognosis (Yang C. et al., 2020). Moreover, inflating immune cells are involved in various steps of antitumor immunity, high infiltration levels of DCs and high-PD-L1-expressing Tregs were reported to be indicators of favorable prognosis in patients treated by ICIs (Wu et al., 2018; Huang et al., 2022). Thus, downregulated immune-related pathways, high infiltration levels of DCs and Tregs may partly explain why patients in cluster B had a higher response to ICIs in our study. However, tumor-infiltrating immune cells are highly heterogeneous. For instance, different DC subsets have significantly different effects on immunity and tolerance in cancer settings (Wculek et al., 2020). The prognosis of colorectal cancer patients with increased numbers of Tregs has also been controversial, which may be attributed to an improper interpretation of heterogeneous FOXP3+ cells as a single population of Tregs (Tanaka and Sakaguchi, 2017). More coming studies based on single-cell sequencing may help elucidate this issue.

The IMbrave150 phase 3 study reported that ICI plus antiangiogenesis-based therapies could improve the survival outcomes of HCC patients over sorafenib alone in the first-line setting, but not all patients can benefit from ICIs (Pinter et al., 2021). Intriguingly, our results suggested that HCC patients in ECM-related cluster A had significantly higher TMB, but almost all HCC patients derived from TCGA database had low TMB. In contrast, patients in cluster B had higher immune and stromal scores. Importantly, we also found that cluster B patients may have a higher response rate to ICIs. Besides, 10 chemotherapy and targeted drugs had great differences in estimated IC50 values between the two molecular subtypes, further indicating that this signature could play a pivotal role in the prognosis and therapeutic responses of HCC patients.

After that, for the convenience of clinical application, we constructed an ECM-related prognostic risk score model using the expression profiles of six genes (SPP1, ADAMTS5, MMP1, BSG, LAMA2, and CDH1), which was internally and externally validated using four independent cohorts. Our results suggested that the expression levels of SPP1, ADAMTS5, MMP1, and BSG were associated with poor prognosis of HCC, while LAMA2 and CDH1 were related to longer survival. Intriguingly, SPP1 overexpression was reported to be associated with HCC progression and immune escape in lung adenocarcinoma (Zhang et al., 2017; Wang et al., 2019). MMP1 and BSG overexpression are also well-known poor prognostic markers of HCC (Liu H. et al., 2021; Ma et al., 2021). High CDH1 mRNA expression was also significantly correlated with better survival outcomes in HCC patients (Wu et al., 2019). However, the prognostic effect of ADAMTS5 in hepatocellular carcinoma remains controversial (Théret et al., 2021). Furthermore, the ECM-related prognostic nomogram demonstrated excellent agreement between the predicted and actual survival outcomes, as well as better prognostic capacity than TNM stage.

In addition to being a prognostic marker, targeting the ECM-mediated immunosuppressive stromal microenvironment and physical barrier in combination with other systemic therapies could promisingly ameliorate the response to those drugs. For example, ECM deposition was related to immunosuppression and gemcitabine resistance in pancreatic cancer (Peran et al., 2021), while the proteolysis of ECM proteoglycans was associated with T cell infiltration in colorectal cancer (Hope et al., 2017). Although various studies targeting different components of the ECM (such as degradation of stromal collagen and hyaluronic acid) have reported desirable success, this strategy has repeatedly failed in clinical studies (Abyaneh et al., 2020), which may be partly explained by the nonspecificity of drugs and complex context-specific roles of the ECM (Mushtaq et al., 2018). Excessive depletion of ECM would compromise or even worsen the outcomes. This was shown in transgenic mouse models exhibiting reduced stromal content. Both fibroblast-depleted tumors and hedgehog-inhibited tumors showed more aggressive behaviors (Rhim et al., 2014; Özdemir et al., 2014). Moreover, excessive removal of ECM components may result in matrix collapse and decreased drug penetration (Abyaneh et al., 2020). Therefore, normalization of the ECM rather than its depletion may be the better goal (Abyaneh et al., 2020). On the other hand, targeting the ECM for drug delivery is another promising therapeutic strategy. For example, matrix-binding ICIs could enhance antitumor efficacy and reduce adverse events in a preclinical melanoma model (Ishihara et al., 2017). However, ECM-targeting drug delivery strategies are mainly explored in animal studies and deserve further validation in clinical trials.

To the best of our knowledge, this is the first and comprehensive study classifying HCC patients based on ECM-related gene expression profiles. Furthermore, an ECM-related prognostic signature and nomogram were developed and validated. However, there were still some limitations in our study, although the prognostic signature was validated by three independent cohorts and demonstrated good accuracy. First, HCC patient classification and the prognostic signature were conducted based on retrospective data, and prospective validation is needed. Moreover, considering ECM components and structures are quite dynamic being subject to protein changes, further proteomics studies are also warranted to better evaluate the impact of ECM on HCC. Second, due to methodological limitations, we were unable to analyze and predict the sensitivity of some essential drugs for HCC patients, such as sorafenib and lenvatinib, which may be addressed in other databases or prospective studies. Third, the candidate genes enrolled in our study were restricted to the ECM-related signature, while the immune microenvironment in tumors is a complex consisting of the ECM and a variety of cells. Thus, the prognostic predictive power of the signature may be limited. Nonetheless, the ECM-related signature provides abundant information about the immune microenvironment and demonstrates good accuracy, which proves to some extent that developing a prognostic model based on an ECM-related signature is rational.

## Conclusion

Collectively, based on ECM-related gene expression profiles, two molecular subtypes in HCC patients were characterized, with distinct clinical outcomes, somatic mutation profiles, and drug sensitivity. Ultimately, an ECM-related prognostic signature was developed and validated. These findings may improve our understanding of the ECM signature in HCC and pave a new path for the assessment of prognosis and drug sensitivity.

## Data Availability

The original contributions presented in the study are included in the article/Supplementary Material, further inquiries can be directed to the corresponding author.

## Supplementary Material

Supplementary Table S1 Association between molecular clusters and clinicopathological variables in HCC patients (*n* = 341).
