# Supplementary material for: Extracellular Matrix-Based Gene Expression Signature Defines Two Prognostic Subtypes of Hepatocellular Carcinoma With Different Immune Microenvironment Characteristics
Source: Front Mol Biosci. 2022 Mar 25;9:839806. doi: 10.3389/fmolb.2022.839806 (PMC8990864; doi:10.3389/fmolb.2022.839806)
Supplement: Supplementary file 1 [file Table1.docx]

Table S1. Association between molecular clusters and clinicopathological variables in HCC patients (n = 341)

|  | **Cluster** | | |  |
| --- | --- | --- | --- | --- |
|  | **Total** | **A** | **B** | **P value** |
|  | **(N=341)** | **(N=273)** | **(N=68)** |  |
| **Age** |  |  |  |  |
| < 60 | 159 (46.6%) | 132 (48.4%) | 27 (39.7%) | 0.253 |
| ≥ 60 | 182 (53.4%) | 141 (51.6%) | 41 (60.3%) |  |
| **Gender** |  |  |  |  |
| Female | 109 (32.0%) | 83 (30.4%) | 26 (38.2%) | 0.274 |
| Male | 232 (68.0%) | 190 (69.6%) | 42 (61.8%) |  |
| **Family history of cancer** |  |  |  |  |
| No | 196 (57.5%) | 156 (57.1%) | 40 (58.8%) | 0.245 |
| Yes | 100 (29.3%) | 77 (28.2%) | 23 (33.8%) |  |
| Unknown | 45 (13.2%) | 40 (14.7%) | 5 (7.4%) |  |
| **TNM stage** |  |  |  |  |
| I | 170 (49.9%) | 135 (49.5%) | 35 (51.5%) | 0.699 |
| II | 84 (24.6%) | 69 (25.3%) | 15 (22.1%) |  |
| III | 83 (24.3%) | 65 (23.8%) | 18 (26.5%) |  |
| IV | 4 (1.2%) | 4 (1.5%) | 0 (0%) |  |
| **Histologic grade** |  |  |  |  |
| G1–G2 | 212 (62.2%) | 173 (63.4%) | 39 (57.4%) | 0.477 |
| G3–G4 | 127 (37.2%) | 98 (35.9%) | 29 (42.6%) |  |
| Unknown | 2 (0.6%) | 2 (0.7%) | 0 (0%) |  |
| **Ishak score** |  |  |  |  |
| 0-4 | 124 (36.4%) | 94 (34.4%) | 30 (44.1%) | 0.068 |
| 5-6 | 74 (21.7%) | 66 (24.2%) | 8 (11.8%) |  |
| Unknown | 143 (41.9%) | 113 (41.4%) | 30 (44.1%) |  |
| **Child–Pugh grade** |  |  |  |  |
| A | 207 (60.7%) | 164 (60.1%) | 43 (63.2%) | 0.414 |
| B-C | 21 (6.2%) | 15 (5.5%) | 6 (8.8%) |  |
| Unknown | 113 (33.1%) | 94 (34.4%) | 19 (27.9%) |  |
| **Vascular invasion** |  |  |  |  |
| Macro | 16 (4.7%) | 11 (4.0%) | 5 (7.4%) | 0.656 |
| Micro | 84 (24.6%) | 67 (24.5%) | 17 (25.0%) |  |
| None | 193 (56.6%) | 155 (56.8%) | 38 (55.9%) |  |
| Unknown | 48 (14.1%) | 40 (14.7%) | 8 (11.8%) |  |
| **Alpha fetoprotein** |  |  |  |  |
| Negative | 87 (25.5%) | 71 (26.0%) | 16 (23.5%) | 0.792 |
| Positive | 254 (74.5%) | 202 (74.0%) | 52 (76.5%) |  |
| **Residual tumor** |  |  |  |  |
| R0 | 301 (88.3%) | 243 (89.0%) | 58 (85.3%) | 0.081 |
| R1-R2 | 14 (4.1%) | 8 (2.9%) | 6 (8.8%) |  |
| Unknown | 26 (7.6%) | 22 (8.1%) | 4 (5.9%) |  |
